# Supplementary figures and images for: Reanalysis of Lactobacillus paracasei Lbs2 Strain and Large-Scale Comparative Genomics Places Many Strains into Their Correct Taxonomic Position
Source: Microorganisms. 2019 Oct 25;7(11):487. doi: 10.3390/microorganisms7110487 (PMC6920896; doi:10.3390/microorganisms7110487)

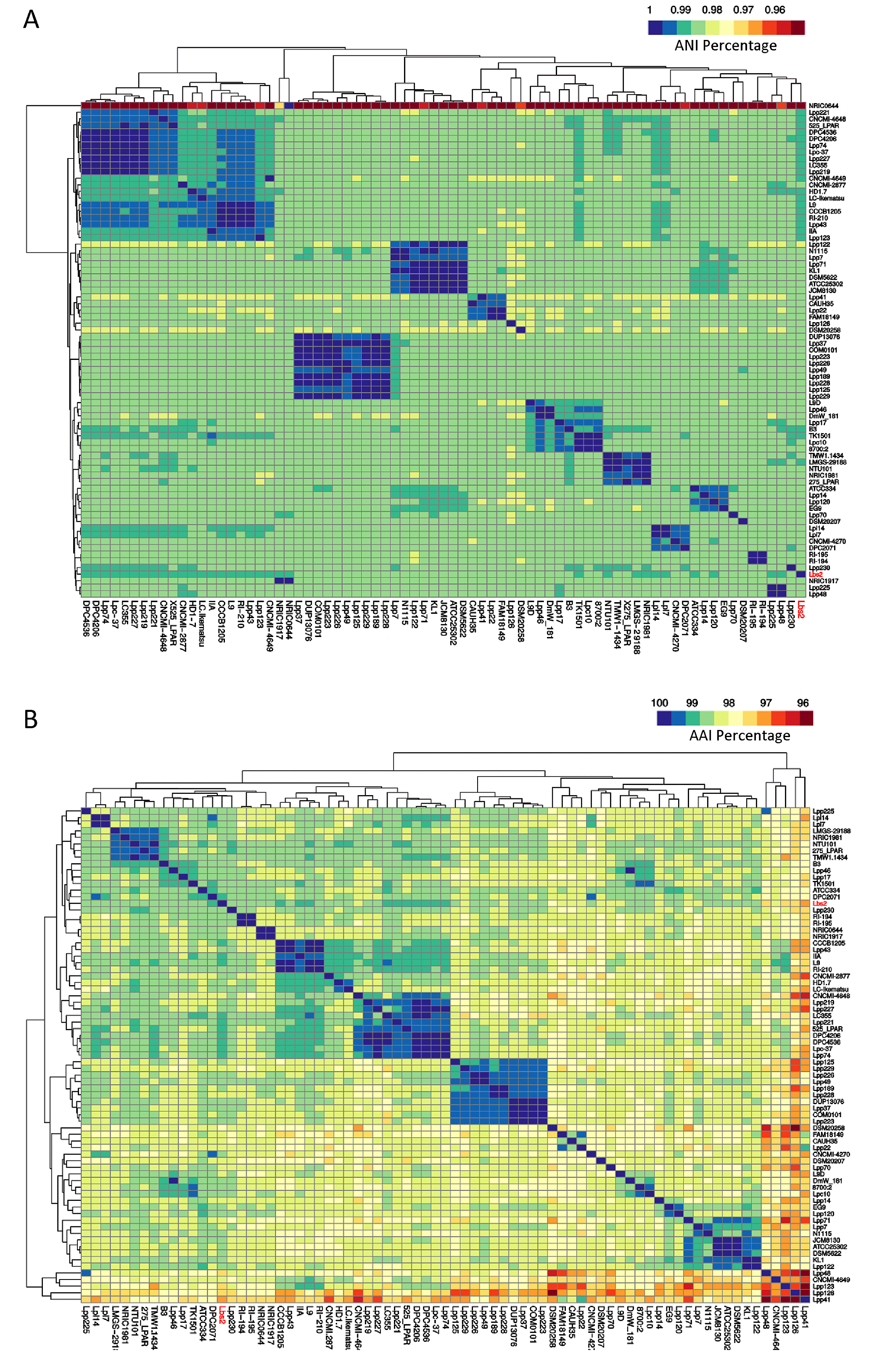

Supplement: Supplementary file 1 [file microorganisms-07-00487-s001.zip › Supplementary-R3/Figure_S1.tif]

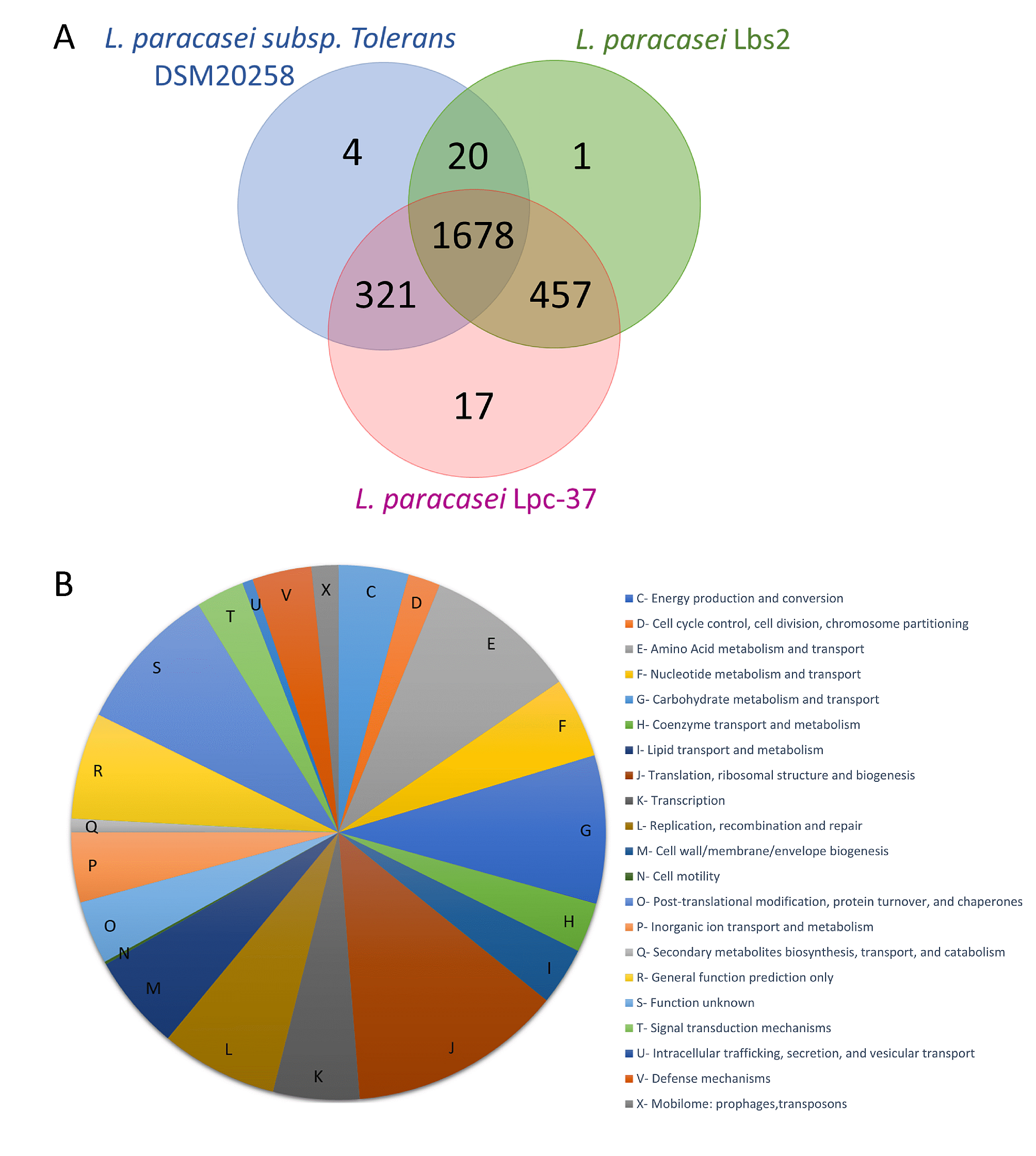

Supplement: Supplementary file 1 [file microorganisms-07-00487-s001.zip › Supplementary-R3/Figure_S11.tif]

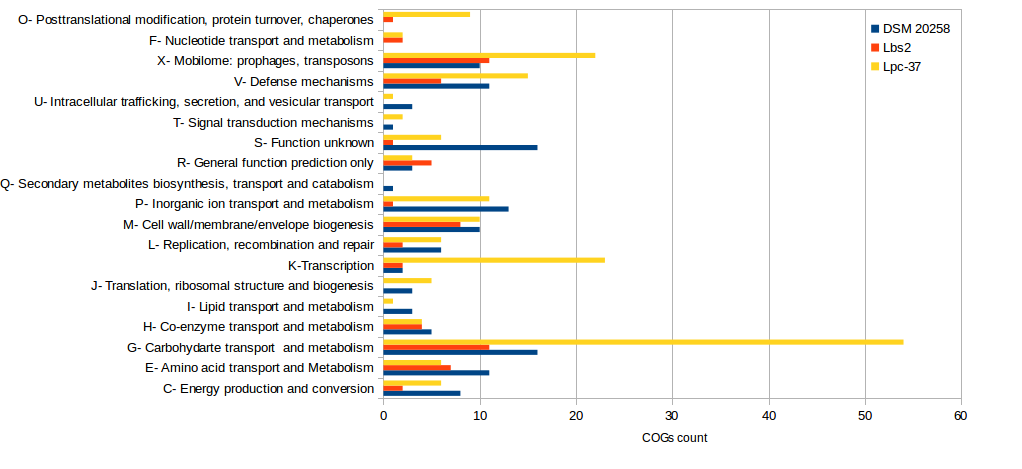

Supplement: Supplementary file 1 [file microorganisms-07-00487-s001.zip › Supplementary-R3/Figure_S12.tif]

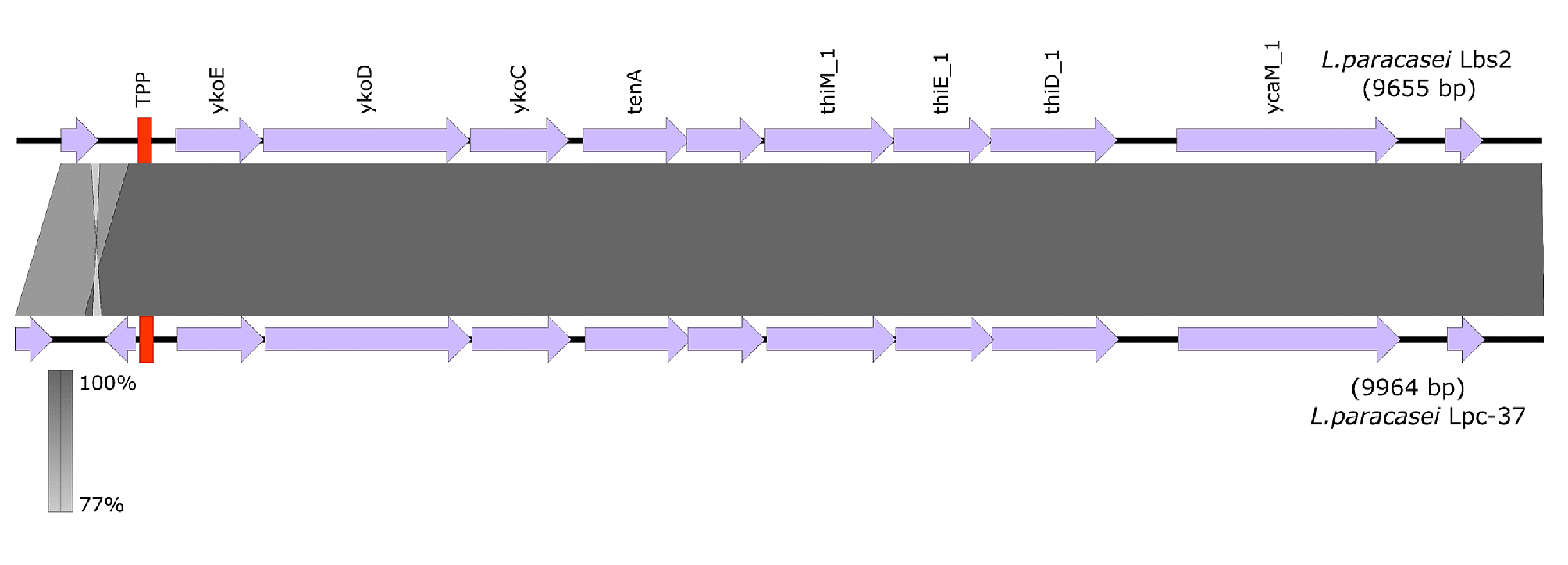

Supplement: Supplementary file 1 [file microorganisms-07-00487-s001.zip › Supplementary-R3/Figure_S14.tif]

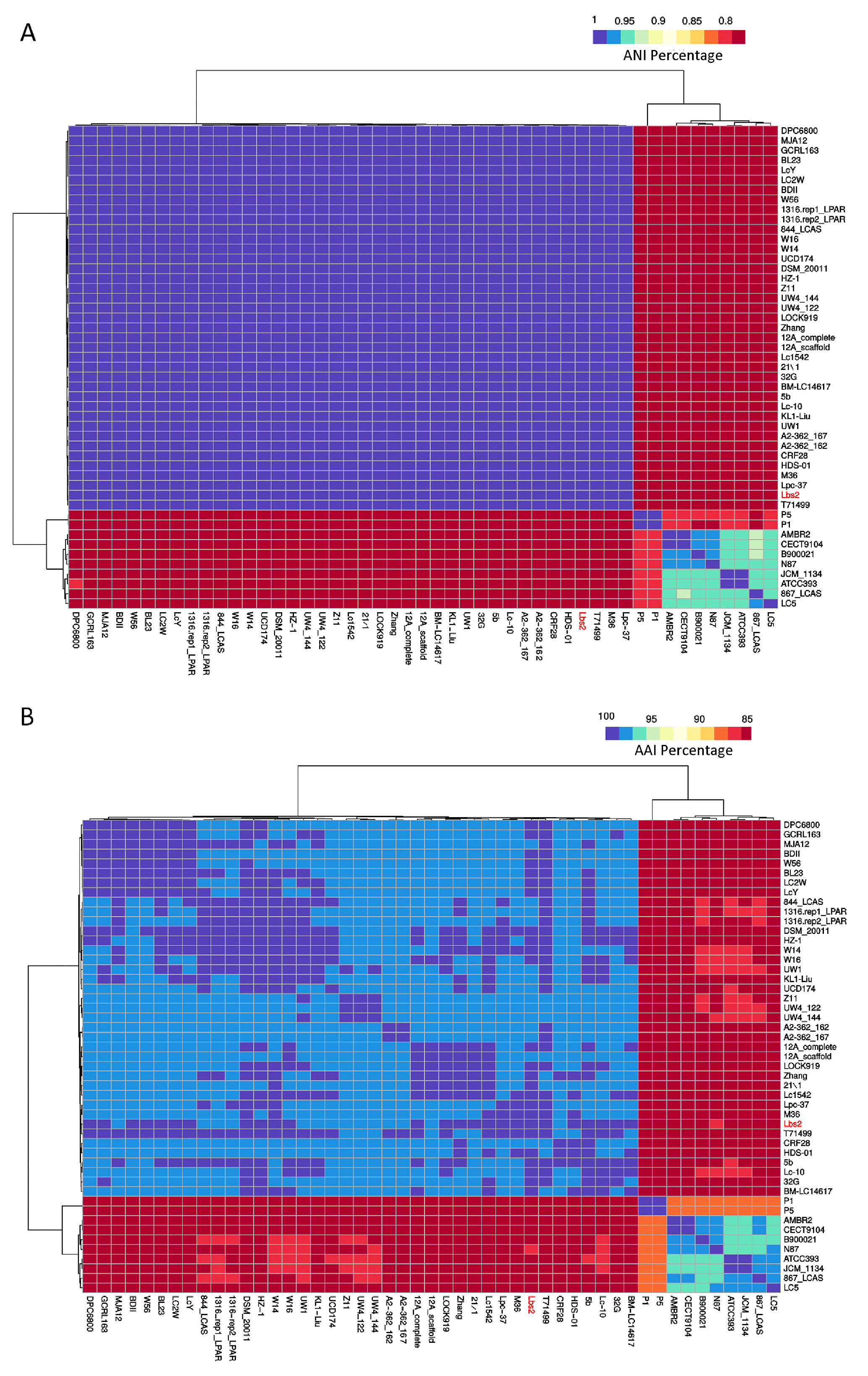

Supplement: Supplementary file 1 [file microorganisms-07-00487-s001.zip › Supplementary-R3/Figure_S2.tiff]

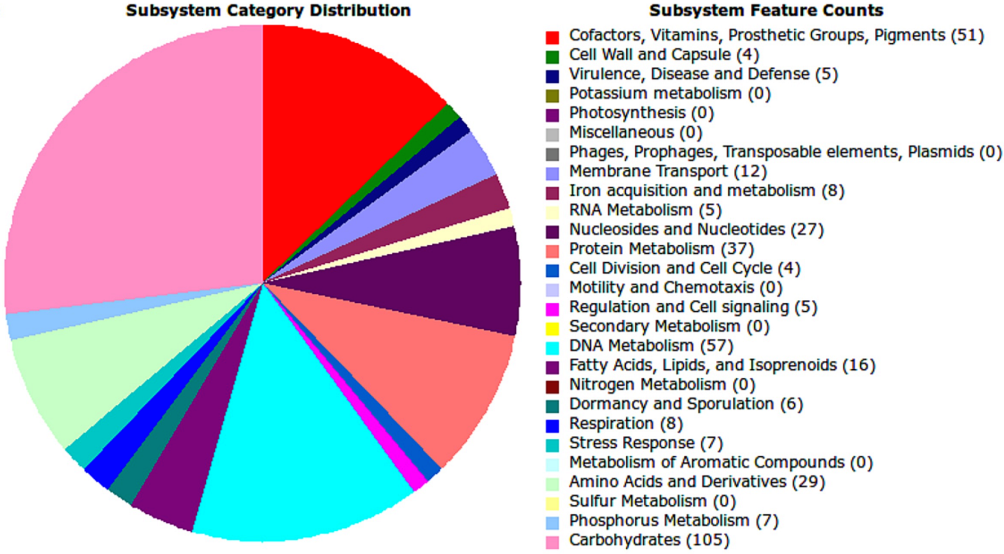

Supplement: Supplementary file 1 [file microorganisms-07-00487-s001.zip › Supplementary-R3/Figure_S3.tif]

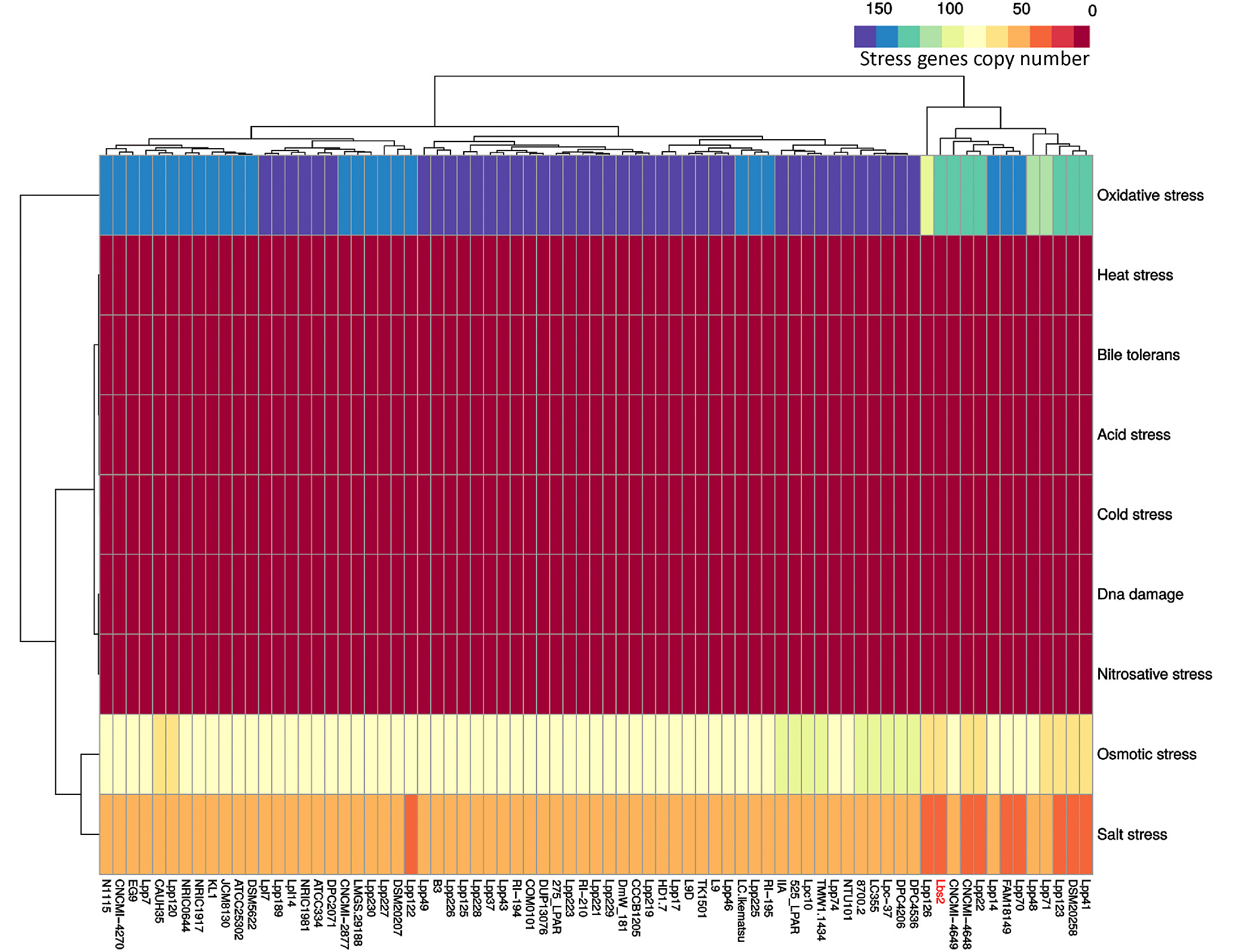

Supplement: Supplementary file 1 [file microorganisms-07-00487-s001.zip › Supplementary-R3/Figure_S6.tiff]

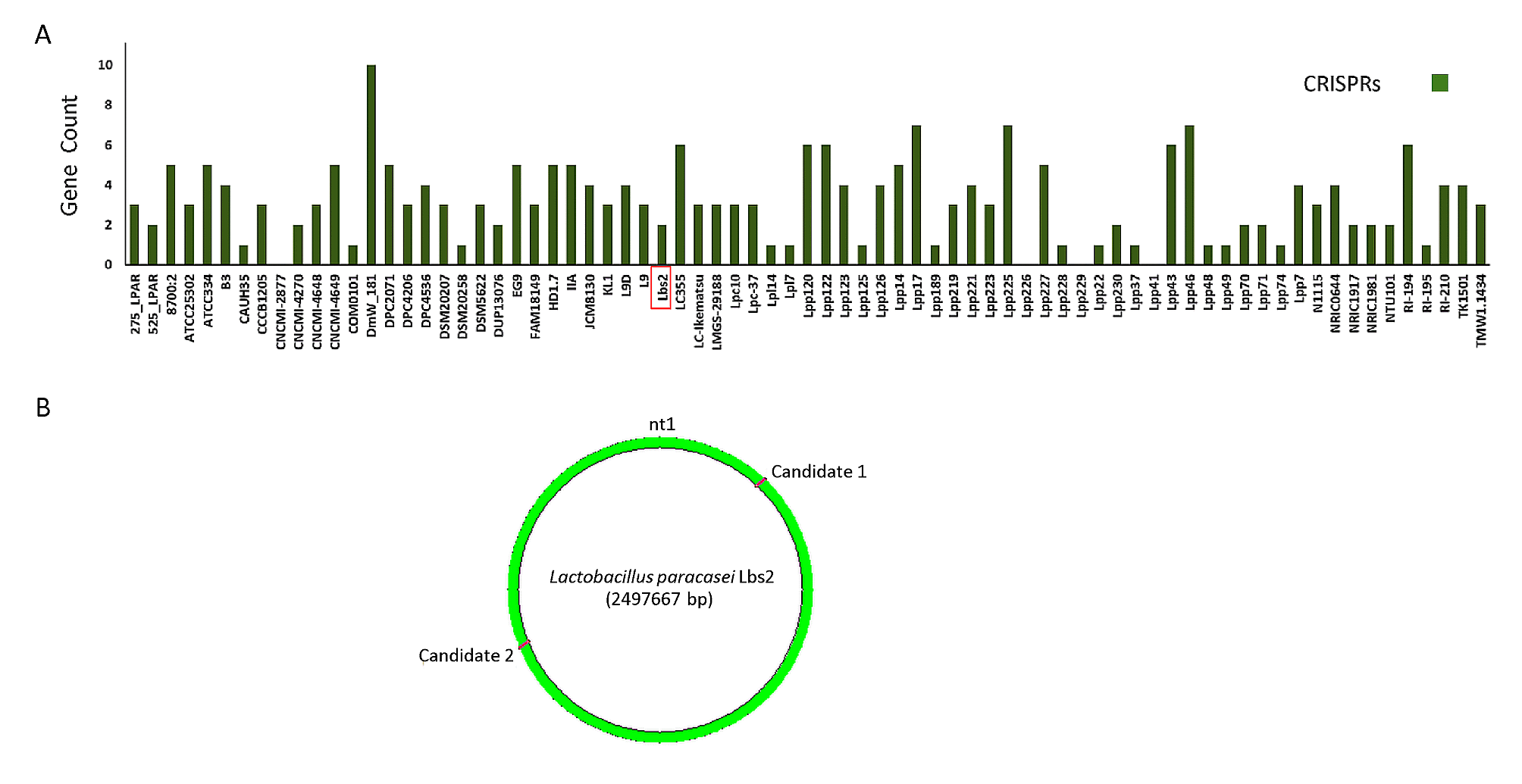

Supplement: Supplementary file 1 [file microorganisms-07-00487-s001.zip › Supplementary-R3/Figure_S8.tif]

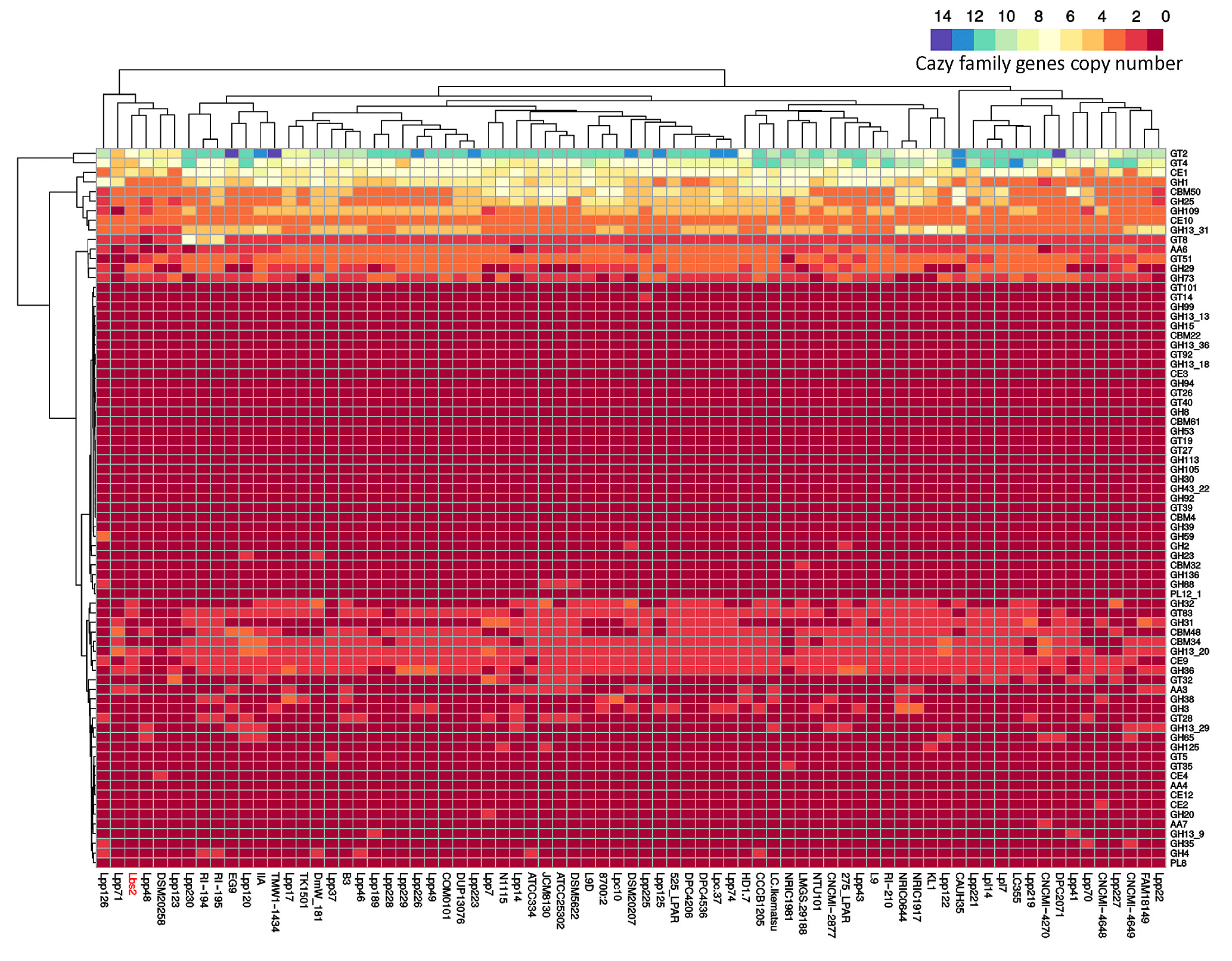

Supplement: Supplementary file 1 [file microorganisms-07-00487-s001.zip › Supplementary-R3/Figure_S9.tiff]
